# Survival analysis of confirmed elephant endotheliotropic herpes virus cases in Thailand from 2006 – 2018

**DOI:** 10.1371/journal.pone.0219288

**Published:** 2019-07-05

**Authors:** Khajohnpat Boonprasert, Veerasak Punyapornwithaya, Pallop Tankaew, Taweepoke Angkawanish, Supaphen Sriphiboon, Chatchote Titharam, Janine L. Brown, Chaleamchat Somgird

**Affiliations:** 1 Center of Elephant and Wildlife Research, Chiang Mai University, Chiang Mai, Thailand; 2 Department of Food Animal Clinic, Faculty of Veterinary Medicine, Chiang Mai University, Chiang Mai, Thailand; 3 Elephant Hospital, National Elephant Institute, Forest Industry Organization, Lampang, Thailand; 4 Department of Large Animal and Wildlife Clinical Sciences, Faculty of Veterinary Medicine, Kasetsart University, Kamphaeng Saen Campus, Nakornpathom, Thailand; 5 Department of Companion Animal and Wildlife Clinics, Faculty of Veterinary Medicine, Chiang Mai University, Chiang Mai, Thailand; 6 Center for Species Survival, Smithsonian Conservation Biology Institute, Front Royal, Virginia, United State of America; Katholieke Universiteit Leuven Rega Institute for Medical Research, BELGIUM

## Abstract

The elephant endotheliotropic herpesvirus (EEHV) has been a known cause of death of young elephants in Thailand for over a decade. In this study, we report on the demography, disease characteristics and mortality of 58 elephants with confirmed EEHV hemorrhagic disease between January 2006 and August 2018 using retrospective data subjected to survival analysis. Median age of EEHV presentation was 29 months, and the mortality rate was 68.97% with a median survival time of 36 h. Most EEHV cases occurred in the north of Thailand, the region where most of the country’s captive elephants reside. The hazard ratio analysis identified application of medical procedures and antiviral medications as being significant factors correlated to the risk of death. Our results indicate a need to focus EEHV monitoring efforts on young elephants and to follow current protocols that advise starting treatments before clinical signs appear.

## Introduction

The elephant endotheliotropic herpes virus (EEHV) is a cause of rapid death in young Asian elephants (*Elephas maximus*) that present with signs of acute hemorrhagic disease or “EEHV-HD” [1,2]. The first report of disease associated with a herpes virus in an elephant was in 1971, and involved pulmonary lymphoid nodules and cutaneous papillomas in an African elephant (*Loxodonta africana*) in Kruger National Park [3]. In 1990, Ossent and others described a lethal case in an Asian elephant that presented with severe hemorrhage in multiple organs, particularly heart, trunk, stomach, and intestine, although the cause was unknown [4]. The discovery of DNA from the causative agent EEHV1 in a 16-month-old female calf in 1999 established it as a novel endotheliotropic herpesvirus specific to elephants [5]. Over the past 20 years, at least 90 lethal EEHV cases in elephant calves worldwide have been reported [6], and it is now considered a cause of high mortality in young Asian elephants in western zoos [7] and throughout Asia [1,8–12]. Despite many reports of confirmed EEHV cases, there is no evidence of epidemic outbreaks of EEHV. Rather, it is considered a sporadic rather than an endemic disease [1,13–15] that affects both Asian and African elephant species [1,10–12,16,17]. The taxonomic classification of EEHV has it in the genus *Proboscivirus*, subfamily *Betaherpesvirinae*, family *Herpesviridae*, order *Herpesviruses* [6]. However, more recently it has been proposed that EEHV should be in a new *Deltaherpesvirinae* subfamily based on the DNA polymerase and glycoprotein B gene [1,7,18]. Seven genotypes of EEHV have been identified so far, with EEHV1A and EEHV1B being the most frequently reported in young calves with acute hemorrhagic disease [7,8,19]. To date, most EEHV studies have focused on viral genetic diversity [8,14,17,20–23], clinical pathology [4,24–27], rapid diagnostic techniques [11,19,28–30], infection confirmation methods [31,32], and treatment protocols [12,33]. Far less attention has been paid to understanding disease prevalence, risk factor identification, epidemiological modeling [34] or changes within the host following infection, all of which are needed to help understand the pathogenesis of this disease.

Collaboration with relevant organizations in Thailand, such as the EEHV Task Force, National Elephant Institute, and Chiang Mai and Kasetsart Universities over the last 10 years has provided a mechanism for data gathering and knowledge sharing that is beginning to shed light on the demographics and clinical characteristics of EEHV in this country, how the elephant responds to infection, and factors influencing disease progression. The aim of this report was to characterize the occurrence of EEHV cases in Thailand between 2006–2018 and analyze data using survival statistical survival models. We believe that this information will provide valuable information to assist in the diagnosis, treatment, and management of this rapidly fatal disease.

## Materials and methods

### Elephants and data collection

We utilized veterinary records of 58 confirmed cases of EEHV in captive elephants reported in Thailand between January 2006 and August 2018, as well as interviews of elephant owners and veterinarians involved with suspected cases. The elephants in this study participated in routine health services and an EEHV surveillance program, which involved several of the authors and was coordinated by the Center of Excellence in Elephant and Wildlife Research, Chiang Mai University (KB, PT, CT, CS) in collaboration with partner institutions: The National Elephant Institute (TA); The Faculty of Veterinary Medicine, Kasetsart University (SS), The Zoological Park Organization; and the Livestock Animal Department of Thailand. Activities are guided by the Thailand EEHV Task Force, chaired by co-author, C. Thitaram. Blood samples were collected by qualified veterinarians associated with the EEHV Task Force from elephants exhibiting signs of EEHV infection at the request of camp owners to confirm diagnosis and arrange treatment, if necessary. Samples were analyzed at the Chiang Mai University Veterinary Diagnosis Center or Kasetsart University Veterinary Diagnosis Center, and all collection procedures were approved by the elephant’s owner and in-charge veterinarian. No blood samples were collected outside of the health care services program for this study. Because only veterinary record data were utilized, Chiang Mai University does not require ethics committee approval.

Data were collected using methods similar to those from studies of Ebola viral outbreaks in humans [35–37], and included demographic information on age, sex, and region of suspected or confirmed EEHV cases. Data on the host response to infection and progression of disease included age (in months) of the first clinical presentation, and the specific time (hour) from clinical presentation to death or recovery. Physical examinations were conducted and based on the Elephant Endotheliotropic Herpesvirus (EEHV) protocol by Cracknell [38] and the Guidelines for Management of Elephant Endotheliotropic Herpesvirus (EEHV) in Asia by Luz and Howard [39]. Clinical signs were recorded as follows: 1) lethargy—a lowered level of consciousness, with drowsiness, listlessness, and apathy; 2) anorexia—lack or loss of appetite; 3) fever—rectal temperature over 38°C; 4) facial edema—excess of watery fluid collecting in the head, neck, and trunk; 5) tongue cyanosis—petechial or patchy hemorrhage starting at the tip of the tongue and moving caudally; 6) diarrhea—loose and watery feces; and 7) bloody diarrhea—red coloration and smell of blood in the feces. Influencing factors included the subtype of EEHV, level (severity) of clinical signs, treatment procedures, antiviral medication (where used), weaning status, and training history that refers to whether elephants were trained for medical intervention by veterinarians, usually with the aid of the mahout. Most untrained elephants were calves less than 2.5 years of age, which limited treatment options, and any potential predisposing factors, such as seasons that were defined by *The Climate of Thailand*, Meteorological Department of Thailand (www.tmd.go.th) and consisted of summer (mid-February to mid-May), rainy (mid-May to mid-October) and winter (mid-October to mid- February) periods or other clinical signs exhibited by the animal. EEHV treatments followed established guidelines [38,39].

### Diagnostic methods

Blood, oral swabs, and tissue samples were collected by veterinarians with the Thailand EEHV Task Force from elephants that presented with signs of EEHV infection for molecular confirmation and EEHV subtype identification. All positive EEHV cases were confirmed by either conventional PCR or qualitative PCR [11,40,41] at the Faculty of Veterinary Medicine, Chiang Mai University or Kasetsart University, Thailand. DNA was extracted using NucleoSpin Blood (Macherey-Nagel, Germany) kits, and samples were screened for EEHV using DNA polymerase (PANPOL) PCR primers [29]. Standard terminase (TER) primers were used for subtype specification of EEHV1A/EEHV1B and EEHV3/EEHV4 [5]. Thirty-five cycles of PCR amplification were completed under the following conditions: 94°C for 1 minute, 50°C for 1 minute, and 72°C for 1 minute followed by 72°C for 7 minutes. The PCR product was visualized on 1% agarose gel stained with ethidium bromide. Positive EEHV TER PCR products were sequenced using an ABI 3700 Automated DNA sequencer (Applied Biosystems, CA, USA) and then analyzed using biological sequence alignment software (Bioedit v.7.2.6). The phylogenetic relationship was generated by multiple alignments of the nucleic acid program (ClustalX). The virus particle was measured by qPCR at Kasetsart University. Although it was not possible to conduct quantitative real-time PCR during the course of clinical signs, blood samples were collected and stored for retrospective testing using EEHV1 and EEHV3/4 specific TaqMan real-time PCR [32]. Samples testing positive for EEHV3/4 are assumed to be positive for EEHV4 and not EEHV3 because EEHV3 is found in African elephants only [18].

### Treatment methods

EEHV treatment protocols were based on established guidelines [38,39] and were as follows: 1) no treatment with sudden death of the elephant; 2) supportive treatment only; and 3) supportive treatment with antiviral medication. Supportive treatments included: i.v. fluid therapy (at least 20 liters per day) with normal saline (0.9%) and dextrose (5%); i.v. antibiotics (e.g., Penicillin G 50,000 IU/kg, S.I.D) intravenously; vitamin C [(ascorbic acid) 6 mg/kg, S.I.D]; and plasma transfusion. Antiviral treatment included acyclovir (12–30 mg/kg B.I.D oral or intravenous) or famciclovir (12 mg/mg B.I.D oral or per rectal) administration.

### Statistical analysis

Demographic and clinical characteristics of EEHV are presented as frequencies and percentages. Survival analyses were conducted using Kaplan-Meier tests and Cox’s regression models [42–44]. Clinical cases included elephants that survived or died within an observation time period, which was set at a maximum of 168 hours (7 days) after onset of clinical signs, and was based on previous studies [1,26,29]. Elephants that were still alive after the survival time or had died from other causes were considered as censored data. Kaplan-Meier analysis was used to estimate survival time of EEHV cases [45,46]. Differences in survival curves for variables were determined by the log-rank test [42]. A Cox’s regression model was applied to estimate the hazard ratio for factors and the occurrence of EEHV. The hazard ratio is an estimate of the ratio of the hazard rate in a treated group compared to controls. In a clinical trial where disease resolution is the endpoint, the hazard ratio indicates the relative likelihood of disease resolution in treated versus control subjects at any given point in time [47]. Because the objective of the analysis was to explore potential factors associated with EEHV, Cox’s regression model was fitted with all variables including sex, subtype, severity, treatment procedure, antivirals, weaning and training [48,49]. All statistical analyses were performed using R statistical software Version 1.1.414 [50].

## Results

### Status of EEHV in Thailand 2006–2018

**Fig 1:**
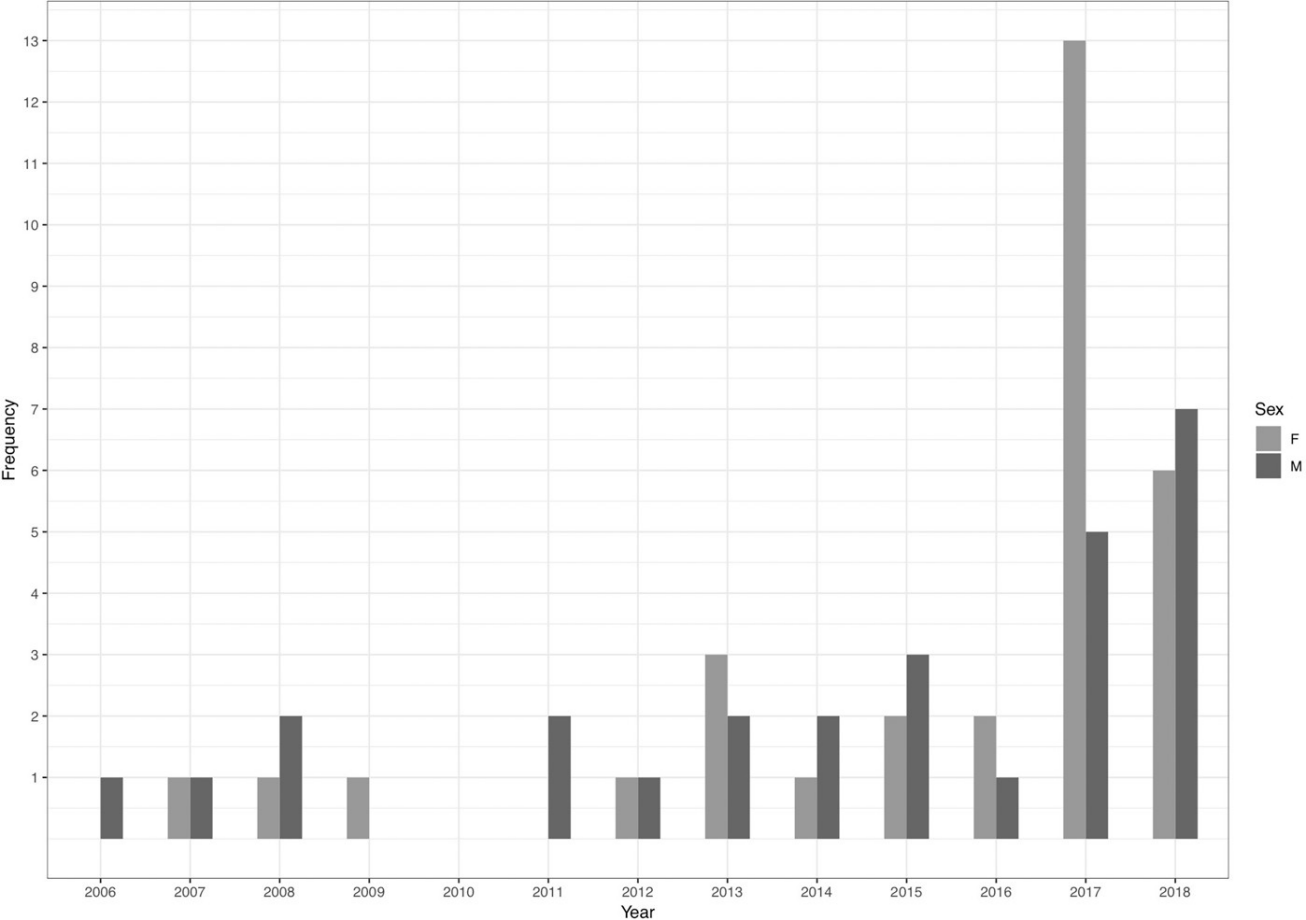
Numbers of confirmed EEHV cases in each year from January 2006 to August 2018, both survivors and non-survivors.

Over a 12-year period, 58 animals were confirmed positive by PCR (Fig 1). The first PCR confirmed case of EEHV occurred in 2006 [11], the first year of this study. Between 2006 and 2016, 1–5 cases were confirmed each year. Since then, the number of cases has increased to over 15 per year, reflecting the increased use of PCR confirmation tests in clinically suspected cases.

### Demographics of EEHV confirmed cases

The demographic characteristics of the 58 cases confirmed by PCR are shown in Table 1. Most cases occurred in elephants less than 2.5 years of age, with a similar distribution between males and females. Cases occurred primarily in the northern part of Thailand, particularly in Chiang Mai province, which had the highest frequency of disease incidence and also the largest captive elephant population. Four EEHV subtypes were identified, with EEHV1A predominating, followed by EEHV4. Two elephants that died were coinfected with EEHV1A and EEHV4.

**Table 1:** Demographic characteristic of 58 confirmed EEHV cases in Thailand between 2006 and 2018.

Characteristics	EEHV cases	%	Survivors	Non-survivors	Fatality rate (%)
**All cases**^a^	58		18	40	68.97
**Sex**					
Female	31	53.45	8	23	74.19
Male	27	46.55	10	17	62.96
**Region**					
North	33	56.90	13	20	60.61
Middle	5	8.62	0	5	100.00
East	1	1.72	0	1	100.00
Northeast	9	15.52	3	6	66.67
West	3	5.17	0	3	100.00
South	5	8.62	2	3	60.00
NA^b^	2	3.45	0	2	100.00
**Subtype**					
EEHV1A	35	60.34	11	24	66.67
EEHV1B	2	3.45	0	2	100.00
EEHV4	19	32.76	7	12	66.67
EEHV1A+EEHV4	2	3.45	0	2	100.00

^a^Age range, months, of all cases (minimum, maximum, median and mean); All cases (3, 216, 29 and 38.47); Survivors (7, 216, 34.5 and 46.61); Non-survivors (3, 114, 29 and 34.71), respectively.

^b^NA = Not available/not known.

### Characteristic features of EEHV confirmed cases

**Fig 2:**
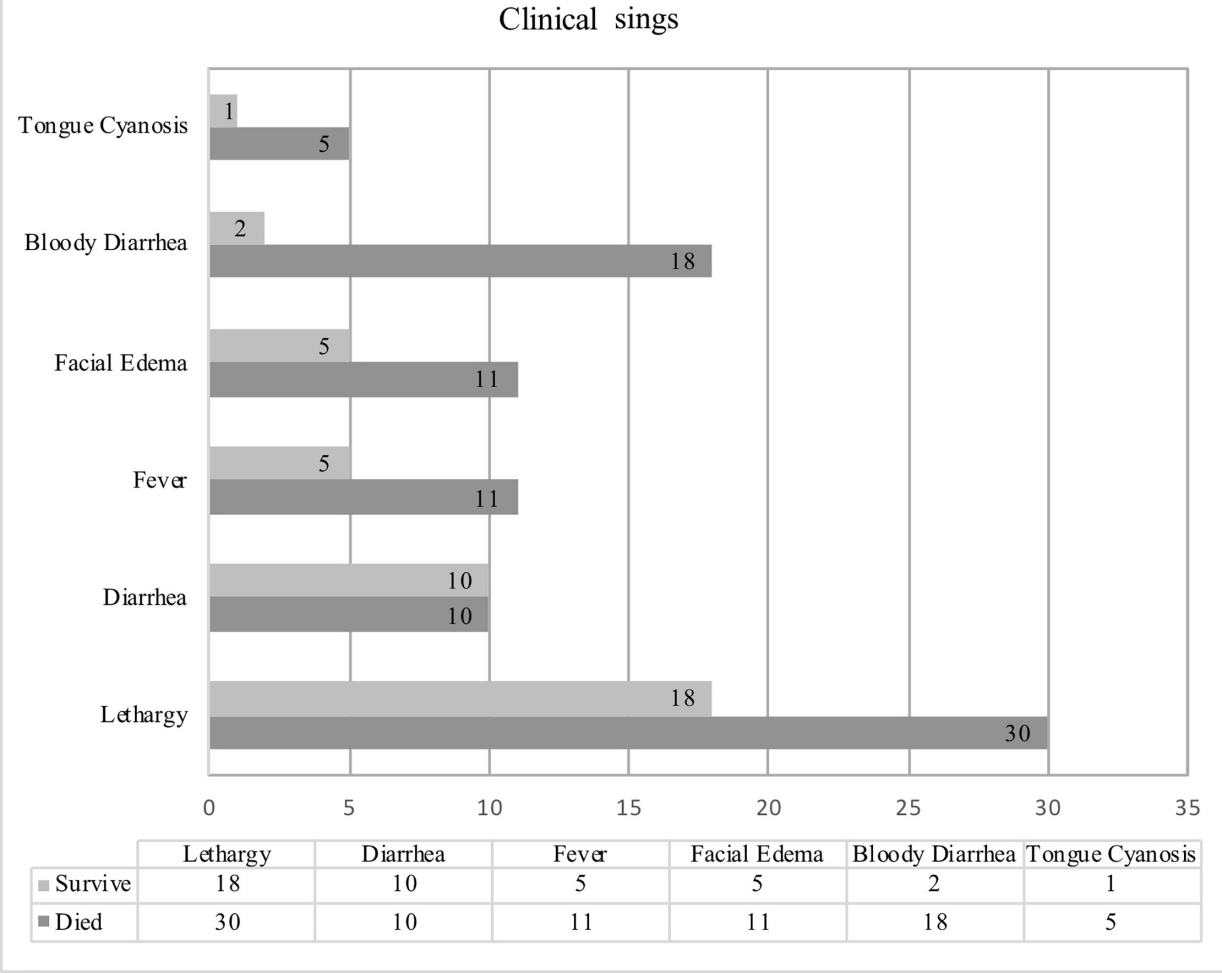
Presentation of clinical features in EEHV confirmed cases.

As shown in Fig 2, 48 of the 58 cases (82.76%) had recorded clinical signs that included lethargy in all cases (100%), bloody diarrhea in 20 cases (41.67%), diarrhea in 10 cases (44.67%), fever in 16 cases (33.33%), facial edema in 16 cases (33.33%), and tongue cyanosis in six cases (12.5%).

### Kaplan-Meier survival curve

**Fig 3:**
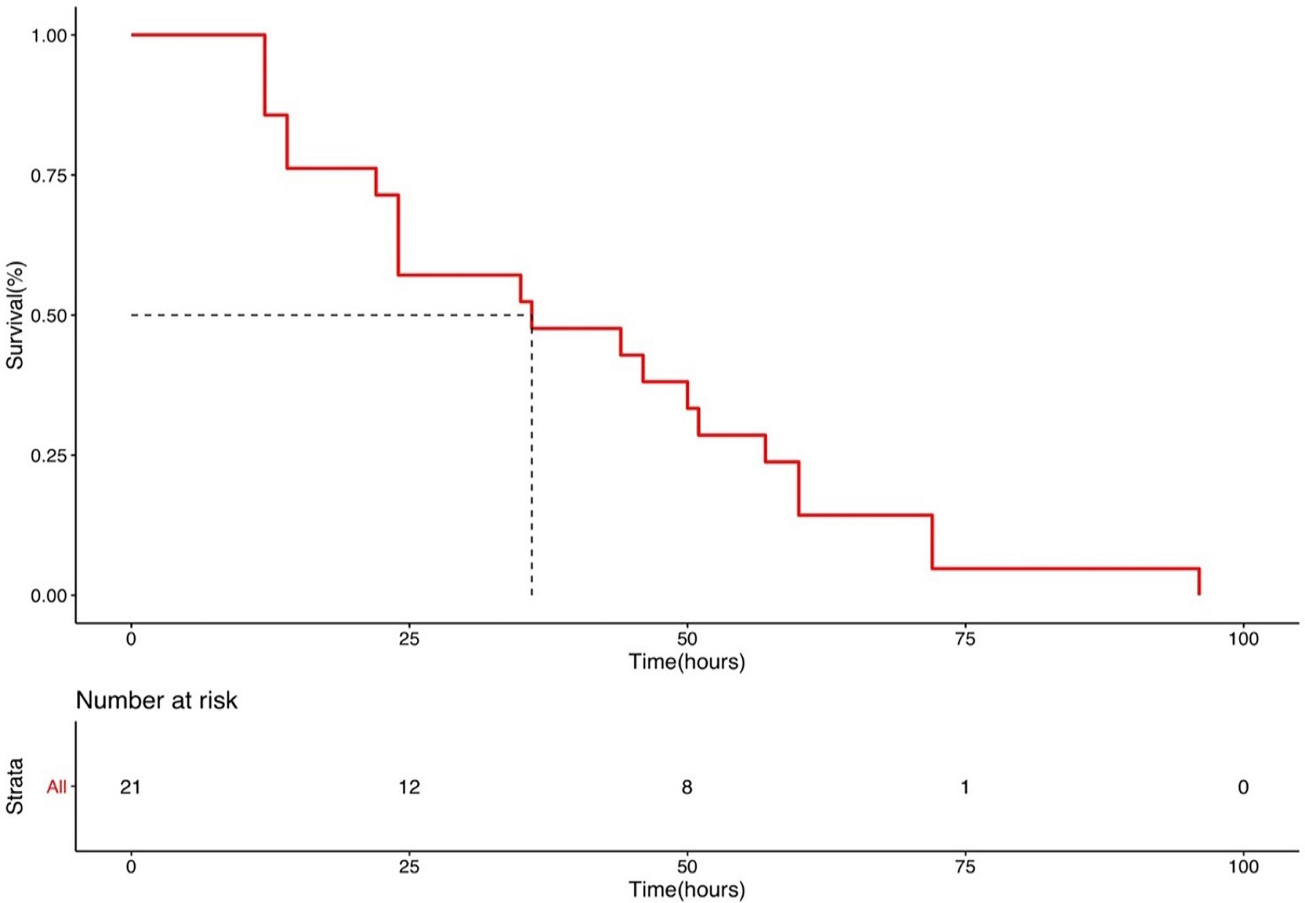
Survival curve by Kaplan-Meier presented median survival time at 36 hours (n = 21).

Of the 58 cases with confirmed EEHV infection, 40 died; the fatality rate of all positive cases was 68.97%. The survival curve was derived from 21 elephants for which there were data from the time of clinical presentation to death, and indicated a median survival time after clinical presentation of 36 hours, by which time 50% of the cases were resolved (Fig 3).

### Comparison of survival curves: Log rank test

Complete data for statistical analysis of each variable from the 58 positive cases were obtained as follows: (1) variables that included sex, subtype, weaning status, training history and season yielded 21 cases with complete data; (2) medical procedures and antiviral therapy yielded 20 cases with complete data. In the univariable analysis, there were no differences in survival curves between levels (categories) of each variable (Table 2); for example, the survival curve between female and male elephants was not different.

**Table 2:** Median survival time and univariable analysis. Data are from 21 cases with complete histories from the time of clinical presentation to death.

Variables	n	Median^a^	0.95LCL^b^	0.95UCL^c^	P-value^d^
Sex					0.9
Female	12	45.0	24	NA	
Male	9	24.0	14	NA	
Subtype					0.9
1 = EEHV1A	11	36.0	22	NA	
2 = EEHV1B	1	24.0	NA	NA	
3 = EEHV4	8	42.5	24	NA	
4 = EEHV1A+EEHV4	1	46.0	NA	NA	
Severity of clinical signs					0.07
1 = Lethargy	1	14.0	NA	NA	
2 = Lethargy+ diarrhea	2	31.0	12	NA	
3 = Lethargy+ bloody diarrhea	9	35.0	22	NA	
4 = Lethargy+ fever + facial edema	6	34.0	24	NA	
5 = Lethargy fever + facial edema+ tongue cyanosis	3	72.0	57	NA	
Medical procedures					0.8
1 = No medication	9	50.0	14	NA	
2 = Supportive treatment only	5	36.0	24	NA	
3 = Supportive treatment and antiviral medication	6	40.0	24	NA	
Antiviral therapy					1.0
1 = No antiviral treatment	10	36.0	14	NA	
2 = Acyclovir	9	36.0	24	NA	
3 = Famciclovir	1	44.0	NA	NA	
Weaning status					0.2
1 = Elephant suckling milk	5	44.0	36	NA	
2 = Weaned elephant	16	29.5	22	60	
Training history					0.7
1 = Not trained	7	36.0	12	NA	
2 = Trained	14	40.5	24	NA	
Season^e^					
1 = Summer (mid-February to mid-May)	6	43.0	12	NA	0.8
2 = Rainy (mid-May to mid-October)	6	40.5	24	NA	
3 = Winter (mid-October to mid-February)	7	24.0	14	NA	

^a^Median of survival time in variables by Kaplan-Meier.

^b^UCL, upper control limit.

^c^LCL, lower control limit.

^d^*p* values were calculated using the Log-lank test.

^e^Seasons were defined by the Meteorological Department of Thailand (MDT).

### Cox’s regression model

**Fig 4:**
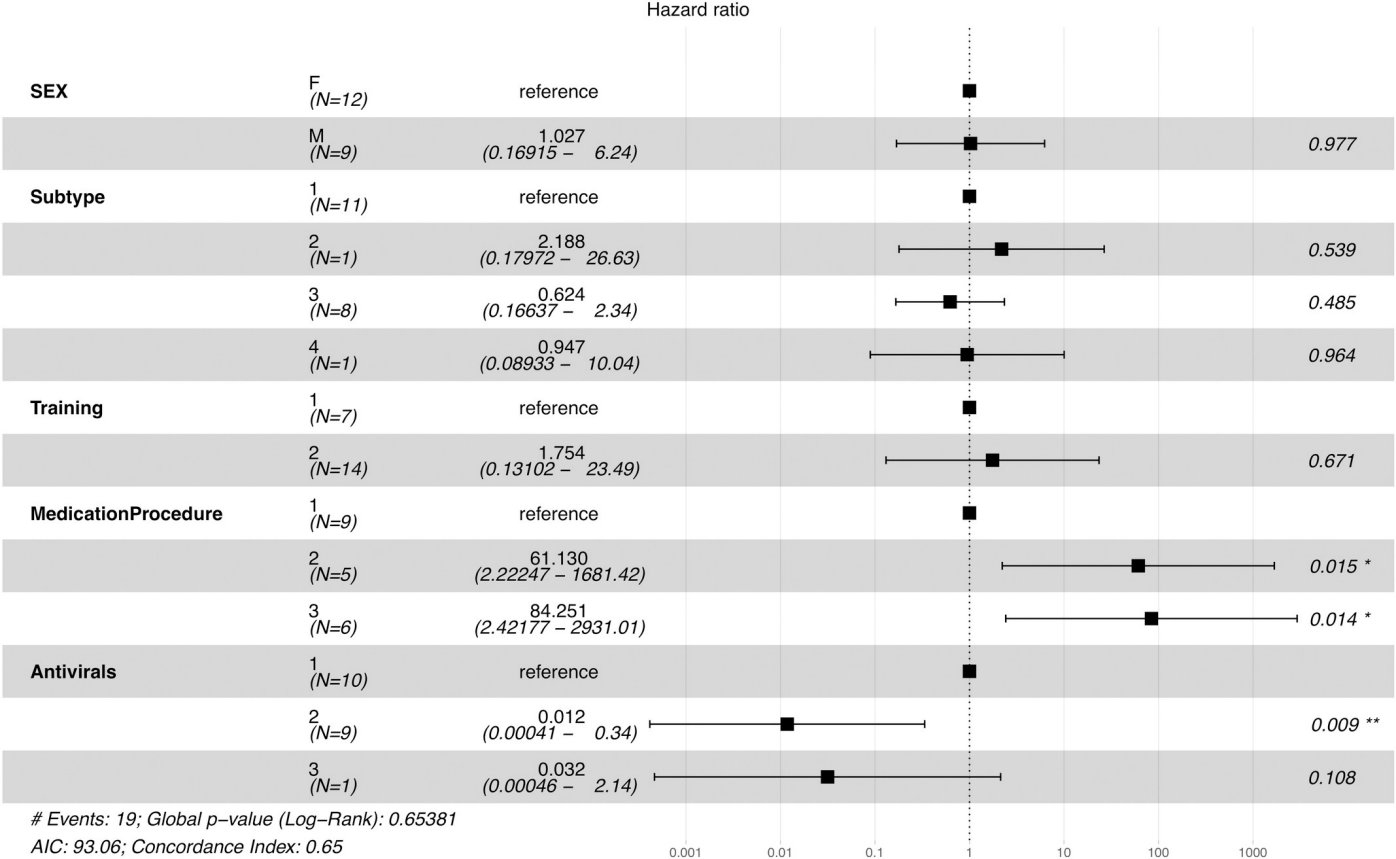
Hazard ratio estimated along with confidence intervals and p-values for each variable.

Cox regression modeling (Fig 4) found the hazard ratio of ‘no medical procedure’ was correlated with a higher risk of death compared to when medical intervention was provided. Moreover, animals that were given acyclovir had a statistically significant decreased risk of death compared with those that received no antiviral therapy. Other factors, including sex, viral subtype, and training were not statistically correlated with the risk of death.

## Discussion

Elephant endotheliotropic herpes virus is a global problem that affects young Asian elephants in a wide range of countries throughout North America, Europe, Asia, and Africa. The virus causes rapid death, especially in young calves [34]. We present here the first report of a retrospective observational study of data collected in Thailand between 2006 and 2018 in an EEHV positive population of captive elephants using a statistical model to describe the response pattern in all confirmed cases. Initially, many young elephants died without being diagnosed, or had clinical signs confused with other hemorrhagic diseases, such as hemorrhagic septicemia. These limitations may have led to relatively low numbers of EEHV clinical cases being identified in neighboring countries as well, such as Cambodia [51], Laos [52] and Myanmar [1]. In Thailand, our data showed that in the first and second quarters of the study, the number of cases was low, presumably reflecting less awareness by any level of domesticated elephant stakeholder in Thailand, e.g. mahouts, elephant owners, camps, veterinarian, academics and researchers. Over the past 15 years, however, EEHV diagnostic techniques, including PCR, have improved substantially [5] and veterinarians are now more capable of diagnosing EEHV infection. In 2016, local and regional EEHV task forces were established in Thailand and Singapore, respectively, to monitor this rapidly fatal disease, provide updated disease occurrences, and share information with field veterinarians involved in elephant care and laboratory testing facilities following published guidelines [39]. Additionally, a systematic recording system was set up to aid in future epidemiological analyses, and laboratory facilities were established to allow for more rapid diagnosis to confirm infection in suspected cases. Last, education about this disease has been enhanced and directed at mahouts, elephant owners, elephant caretakers and camp managers to improve reporting of suspected EEHV cases. Thus, the increase in confirmed cases during the last 2 years of the study is presumed to reflect improvements in disease recognition and diagnosis, especially through the use of PCR. However, we must acknowledge that there are as yet no empirical data to support this assumption, so we cannot rule out the possibility that there also may have been an actual increase in the incidence of the disease over this time period.

Several studies have reported clinical signs, presentations and outcomes following EEHV infection in other range counties [51–53], with results similar to ours. The survival time in the present study was only 1.5 days, which agrees with previous reports that fatality occurs after clinical signs are present for 1–7 days [29,54]. The mortality rate in the present study was 70%, not unlike the fatality rate of 80% in North America [9,29]. Descriptive analysis of demographic outcomes showed a high incidence of EEHV in northern Thailand, which also is the region with the highest numbers of captive elephants [55]. The most common EEHV subtype was EEHV1A, which agrees with findings in North America, Europe, India and Thailand [10,34,54,56,57]. Testing for EEHV5 by PCR was not performed because we lacked the specific primer in our laboratory, but this subtype is known to occur in Asian elephants [41,58,59] and should be investigated. Clinical signs in our study also reflected the findings of others, with the most frequent being lethargy, fever, facial edema, and tongue cyanosis [2,34,52].

According to EEHV treatment guidelines, antiviral drugs should be given in suspected EEHV-HD cases. Acyclovir is commonly used and is easier to obtain than famciclovir in Thailand. There were several clinical cases in this study where acyclovir was not administered because of sudden death of the calf. But others that were treated with antiviral drugs, antibiotics and supportive fluids did survive. Fluid therapy is viewed as an important part of EEHV-HD treatment [60]. Intravenous fluids are supportive by maintaining intravenous volume and electrolyte balance, protecting against hypovolemic shock [12]. Crystalloids (0.9% normal saline, ringers etc.) as an i.v. bolus (0.3–4 mL/kg) followed by rectal fluids is now recommended [61] and has been used in successful treatment of EEHV-HD cases. In addition, plasma and colloids may also be used to support oncotic pressure [12].

We used survival analysis of descriptive data, as described for medical analyses of “time to an event” data [44]. In the present study, a key event was the time from EEHV clinical presentation to death. The analysis allowed us to examine changes over time and factors that influenced survival, which are critically important in understanding host responses and the progression of the disease to survival [43]. Analysis of survival data frequently uses the Kaplan-Meier method, the Cox proportional hazard model, and the log-rank test to generate survival curves, test differences among survival curves for each variable, and determine risk factors [62]. In our study, survival times for each variable showed no statistically significant differences; for every variable, the disease pattern progressed the same over time.

Results from the Cox’s regression model showed that two factors influenced survival time: medical procedures and antiviral therapy. Some elephants that received intensive care still died, possibly because those cases presented with high severity signs or treatment was not initiated in time. As expected, antiviral use reduced the risk of death in accordance with several other reports that some young affected elephants survive through the use of antiviral medication, namely acyclovir or famciclovir [1,12,33]. Information from statistical models can inform and offer guidance for increased intensive monitoring and provision of better management procedures for elephants at risk, as well as indicate the need for future studies. For example, it has been suggested that stress may increase the risk of EEHV-HD by compromising the immune system [56,57,63]; however, there are as yet no empirical data to support that theory, so it should be explored.

According to EEHV Task Force guidelines, young calves under the age of 10 years should be closely monitored. Today, the recommended protocol is to begin treatment before clinical signs are observed, otherwise it is usually too late. Thus, all calves should be monitored routinely, although this requires real-time PCR, which is not always available. Thus, more support is needed to improve elephant mobile veterinary services, increase the number and capacity of diagnostic laboratory facilities, and educate elephant owners, camp managers, elephant curators, veterinarian assistants and mahouts about this disease and how to monitor and treat it. Data on confirmed EEHV cases also should be included in a global database so that future statistical analyses can be conducted to provide epidemiological data related to EEHV, as has been done with studies of Ebola [35–37]. For example, to identify risk factors associated with EEHV-HD, information on husbandry and management, weather conditions, weaning, and nutrition, health and immune status, should be routinely collected.

The limitations of the present investigation were the retrospective study design, missing information on some variables, and the relatively small number of cases. For 37 cases, the data were incomplete because initially we lacked knowledge about EEHV pathogenesis, including clinical signs, diagnostic capability and adequate data recording. Moreover, some cases occurred rapidly in remote areas of the country or in elephant camps where access made it difficult to collect samples or interview mahouts to get accurate information. Nevertheless, this study is significant as it provides the first assessment of factors related to survival in EEHV-infected elephants.

## Conclusions

The findings from this study indicate that infected elephants had a median survival time of only 36 hours. Factors that were linked to this short survival time indicate the urgent need for intensive observation and prompt treatment to reduce case mortality. As diagnostic capabilities improve in elephant range countries like Thailand, more intensive monitoring will be possible, allowing veterinarians to treat cases more effectively.

## Supporting information

S1 Table All information of 58 confirmed EEHV cases during January 2006 –July 2018. (XLSX)
